# The role of self‐compassion and compassion toward others in burnout syndrome in a sample of medical students

**DOI:** 10.1002/pchj.692

**Published:** 2023-10-31

**Authors:** Ignacio Ramos‐Vidal, Érika Ruíz

**Affiliations:** ^1^ Departamento de Psicología Social Universidad de Sevilla, Facultad de Psicología Sevilla Spain; ^2^ Facultad de Psicología Universidad Pontificia Bolivariana Monteria Colombia

**Keywords:** burnout, cluster analysis, compassion fatigue, compassion toward others, medical students, self‐compassion

## Abstract

Burnout produces negative effects on academic performance, and, in turn, feelings of inefficiency that are detrimental to students' psychosocial well‐being. The aim of this research is to determine the effects that self‐compassion and compassion toward others have on various burnout dimensions in a sample of medical students. A total of 235 medical students (61.7% women) aged between 16 and 32 years old (*M* = 19.82; *SD* = 2.37) belonging to a Colombian university participated. A cluster analysis to segment the population according to burnout was carried out along with nonparametric contrasts to identify differences in the levels of self‐compassion and compassion toward others between each profile. A series of regression analyses was designed to find out how each type of compassion was associated with burnout on each profile. The cluster analysis allowed us to identify three profiles. The low‐involvement profile (*n* = 51) is characterized by low depersonalization, intermediate levels of emotional exhaustion and personal accomplishment and exhibits low levels of self‐compassion and compassion fatigue compared with the other profiles. The positive‐adaptation profile (*n* = 104) is characterized by low depersonalization levels, intermediate degrees of emotional exhaustion and high levels of personal accomplishment and exhibits the highest levels of self‐compassion and compassion fatigue compared with the other profiles. The high‐demand profile (*n* = 104) is characterized by intermediate depersonalization levels, medium–high levels of emotional exhaustion and high levels of personal accomplishment and exhibits intermediate levels of self‐compassion and low levels of compassion fatigue. Establishing profiles based on burnout allows students to be segmented and for precise knowledge to be acquired about the effects that both types of compassion have on the dimensions of burnout.

## INTRODUCTION

The syndrome of being burnt out because of work (*burnout*) consists of a prolonged response that subjects experience in relation to emotional and interpersonal stressors in the work context (Maslach & Leiter, 2016). The first investigations of the construct emerged in the early 1970s (Freudenberger, 1974) to describe the physical and emotional exhaustion process experienced by physicians as a result of their continuous interactions with patients (Maslach, 1978). Subsequently, the first measurement instrument appeared (Maslach & Jackson, 1981), which has been progressively perfected (Maslach et al., 1997) and adapted to evaluate the syndrome in different contexts and populations (Cordoba et al., 2012; Faye‐Dumanget et al., 2017).

Burnout is a multidimensional construct made up of the factors of depersonalization, emotional exhaustion, and the feeling of inefficiency and low personal accomplishment (Maslach & Schaufeli, 2018). Depersonalization describes the extent to which workers keep a cold and distant attitude toward the users they serve. Emotional exhaustion is characterized by a state of fatigue as a result of work and a lack of ability to meet the users' demands. Feelings of inefficiency and low personal accomplishment reflect the degree to which workers experience a low level of perceived competence in task execution, which leads to reduced self‐efficacy.

Forty years of research on this syndrome has shown that burnout produces harmful effects on the physical and mental health, productivity, and psychosocial well‐being of workers (Maslach & Leiter, 2016), and that these consequences radiate into the family environment (Kocalevent et al., 2020). The literature suggests that interpersonal relationships and being in permanent contact with users are the main burnout precursors (Maslach, 1978). Therefore, the professions in which there is a higher prevalence of burnout are those linked to education and the provision of health services. A recent systematic review showed that the burnout prevalence in university professors is 37% (Fernández‐Suárez et al., 2021), while in health workers, in the most conservative studies, the figure fluctuates between 19.6% for workers in palliative care units (Parola et al., 2017) and 39.3% for primary‐care professionals (Navarro‐González et al., 2015). The common denominator in both professions is that the professional practice of health professionals and teachers requires maintaining continuous contact with patients or students.

### Burnout syndrome in medical students

In recent years, special attention has been paid to characterizing burnout syndrome in university students. This syndrome in the educational environment is termed academic burnout, and, in essence, consists of equating the effects that activities related to studies cause in students with the consequences associated with exposure to the emotional and interpersonal stressors that workers experience in the work context (Caballero et al., 2010). Burnout study in the academic context becomes especially relevant if the negative impact that this phenomenon produces on the students' academic performance is considered, along with the harmful effect that the low‐performance perception produces on their psychosocial well‐being (Richardson et al., 2012). A recent meta‐analysis that included 29 investigations representing a sample of more than 100,000 students identified the existence of a negative and strong relationship between burnout and academic performance (Madigan & Curran, 2021). The research concluded that burnout causes negative effects of moderate intensity on academic performance (*r*_*c*_^+^ = −.24), with the feeling on the inefficiency dimension being the one that has the strongest effects on academic performance (*r*_*c*_^+^ = −.39).

The study of burnout in university students in the area of health, mainly nursing and medicine, has received considerable attention in recent years (Boni et al., 2018; Wang et al., 2019), owing to the effects that the syndrome produces on both academic performance and the physical and mental health of the students. In these careers, the peculiarity lies in the fact that, as part of the training process, students have to come into contact with patients, making them susceptible to suffering from the syndrome without the need to be active professionals. Students who undertake health studies are exposed to additional stressors given that: (a) they must respond to academic demands; (b) they simultaneously, and as part of their internships, stay in contact with patients and, to a lesser extent, with the patients’ relatives; and (c) unlike active professionals with a consolidated career, they often lack the experience and coping resources necessary to respond to the demands that professional practice entails.

Previous studies have applied classification methods to segment medical student populations. Dahlin and Runeson (2007) conducted a 3‐year follow‐up prospective study with medical students. The first profile (*n* = 46; 47%) was characterized by high levels of burnout, while the second profile (*n* = 52; 53%) presented low levels in the three dimensions that characterize the syndrome. In another proposal, Kusurkar et al. (2021) applied cluster analysis to detect subgroups based on burnout levels in a sample (*N* = 990) of PhD medical students. This analysis allowed the authors to identify three subgroups according to burnout level. Their results showed that the levels of motivation and engagement were higher in the subgroup characterized by lower levels of burnout.

The effects that burnout have in students of health specialties are similar to those identified in active workers. In the case of medical students, the appearance and incidence of the syndrome tend to worsen in the final term of studies, coinciding with a residency, which involves frequent contact with patients. Nonetheless, the appearance of burnout in medical students is not restricted to the final term of their training. Several studies identify a high prevalence of the syndrome before starting residency (Frajerman et al., 2019), and even during the first years of study (Boni et al., 2018). The results concerning the prevalence of burnout syndrome in medical students show unclear results. A recent meta‐analysis that included results from 3006 investigations (Erschens et al., 2019) showed a prevalence that ranged between 7% and 75.2%, depending on the country, measurement instruments used, and evaluated symptoms. On the other hand, a meta‐analytical review (Frajerman et al., 2019) that included 24 studies, representing a total sample of 17,431 students, found that 44.2% of this population exhibited levels of burnout considered as posing a high risk for health. The results of the examined studies show the magnitude of the phenomenon and the need to ascertain the role played by other psychosocial processes in burnout activation. There are several processes associated with burnout in the health professions and in students of care professions. Two processes that have received increased attention in recent years relate to sentiments of compunction experienced when people witness the pain of others. The next section is devoted to explaining the role of compassion fatigue and self‐compassion as triggering factors associated with burnout syndrome in medical students.

### Compassion fatigue, self‐compassion, and burnout syndrome

One of the factors shown to aggravate burnout symptoms in health professionals is the so‐called compassion toward other people, also called compassion fatigue, which is considered a form of vicarious trauma (McCann & Pearlmann, 1990) and is caused as a result of exposure to situations of suffering and of feeling the pain of others. Gilbert et al. (2017) define compassion as sensitivity to suffering in the self and others with a commitment to try to alleviate and prevent it. Compassion fatigue derives from the feeling of compassion toward others and is identified as a type of secondary traumatic stress, characterized by the emission of responses, behaviors, and emotions that appear when individuals are aware of a traumatic episode experienced by a loved one or by the psychosocial effects derived from helping or wanting to help a person who suffers (Figley, 2002). The literature shows that compassion toward others, while it is considered a form of secondary traumatic stress, can produce consequences similar to those produced by exposure to primary traumatic stress, including the appearance of anguish, avoidance behaviors, cognitive changes, or functional impairment (Figley, 1995).

Compassion toward others and self‐compassion can be considered two sides of the same coin. The first occurs owing to exposure to the suffering of others (Figley, 1995, 2002), while compassion toward oneself consists of not avoiding and remaining open to one's own suffering, without trying to disconnect from it, and generating the desire to alleviate the suffering (Neff, 2003). Recently, research on compassion toward others and toward oneself has enjoyed a growing momentum, especially in relation to health professionals (Montero‐Marin et al., 2020). This is because several studies show connections between both types of compassion and burnout syndrome in health professionals (Zhang et al., 2018). Recent studies also suggest that both self‐compassion and compassion fatigue are highly prevalent in medical professions (Ondrejková & Halamová, 2022).

A cross‐sectional study carried out on a sample of 440 primary‐care workers (Montero‐Marin et al., 2020) showed that self‐compassion explained between 5% and 18% of the variability of the burnout dimensions, with the self‐judgment dimension being the one that causes higher‐intensity effects on burnout (Beta = .36; *p <* .001). In a meta‐analysis including 15 studies representing a sample of 900 nurses working in oncology, compassion fatigue was identified to be present in workers who reported medium–high burnout levels (Ortega‐Campos, Vargas‐Roman, Velando‐Soriano et al., 2019). A correlational meta‐analysis developed by Zhang et al. (2018) including data from 11 studies, representing a sample of 4054 nursing professionals, identified a positive and strong correlation (*r* = .59) between compassion fatigue and burnout.

The growing number of studies addressing the relationship between compassion toward others, self‐compassion, and burnout in active health professionals contrasts with the few studies that address this phenomenon in health‐degree students. A study carried out with a sample of 103 British women who were studying midwifery (Beaumont, Durkin, Hollins et al., 2016, p. 19). The results showed a positive and broad intensity relationship between compassion toward others and burnout (*r* = .55; *p* < .01), and a moderate inverse correlation between self‐compassion and burnout (*r* = −.31; *p* < .01). Furthermore, a comparative study carried out on a sample of 422 nursing students showed that the compassion levels toward others and burnout in students in final courses were significantly higher than those reported by early courses students (Michalec et al., 2013).

Compassion fatigue, both toward others and toward oneself, is related to burnout syndrome in several ways. Some studies have found that compassion fatigue intensifies burnout syndrome by reducing concentration and attention and hindering communication patterns between health professional or medical students and their patients (Spickard Jr et al., 2002). On the other hand, previous research has shown that self‐compassion produces a protective effect against developing burnout syndrome. A prospective cohort study with a sample of 807 pediatric residents conducted by Kemper et al. (2019) showed that self‐compassion at Time 1 was negatively related to burnout and stress at Time 2 (*β* = −0.17; *p* < .05). In line with this, a recent study with a sample of physicians, nurses, and medical residents in Lebanon showed that self‐compassion is negatively related to all burnout dimensions (Hashem & Zeinoun, 2020). On the other hand, knowing the possible differential effects of both kinds of compassion fatigue on burnout could be helpful in empirically determining the roles that both exert on burnout syndrome. Although previous studies suggest that self‐compassion and compassion toward others are the effects from knowing about a traumatizing experience that a person has suffered (i.e., Figley, 1995), the effects on mental health and well‐being could differ. For example, some recent studies suggest that self‐compassion may act as a protective factor against burnout (Martínez‐Rubio et al., 2021; Pereira et al., 2022), other investigations evidenced that self‐compassion exacerbated burnout (Kemper et al., 2019), while still other studies identified the moderating role of self‐compassion between burnout syndrome and compassion fatigue (Galiana et al., 2022). Even though in general terms the studies are consistent, showing that compassion toward others aggravates burnout syndrome (Ortega‐Campos et al., 2019), recent studies suggest that a certain degree of compassion fatigue may reduce burnout (Gerber & Anaki, 2021). Considering the relative inconsistency of the literature reviewed, identifying the differential effects of the two compassion types on burnout syndrome could be a contribution to the existing literature.

On the other hand, previous research has demonstrated empirical connections between self‐compassion and compassion fatigue and burnout syndrome. In this regard, Beaumont et al. (2016) evaluated a sample of student counsellors and student cognitive behavioral psychotherapists (*n* = 54) in their final year of study, finding that self‐compassion acts as a protective factor against burnout syndrome. Their results also evidenced that compassion fatigue and burnout are common phenomena during the final stages of educational processes and that their effects tend to be less harmful when students receive training [for example: Mindfulness‐Based Stress Reduction (Jiménez‐Gómez et al., 2022) or Compassionate Mind Training (Gilbert, 2009)] to prevent and manage the symptoms that both cause (Beaumont et al., 2016).

Although there are few studies that analyze the effect that the two types of compassion have on burnout in students studying for health degrees, multiple intervention proposals have been identified to effectively manage compassionate feelings (Klein et al., 2018; Tucker et al., 2017). Thus, although there is no extensive empirical evidence confirming the relationship between the two types of compassion and burnout in health‐career students, the expansion of interventions addressing this issue indicates that it is a relevant phenomenon for psychosocial well‐being.

One conclusion drawn from the reviewed literature is that research focuses on studying the prevalence of compassion toward others and self‐compassion based on the burnout levels (medium‐high‐low) presented by the evaluated population (Ortega‐Campos et al., 2019). However, considering that burnout is a multidimensional construct, whose factors can exhibit a heterogeneous behavior, the application of cluster analysis (Vuik et al., 2016) can be an effective alternative to detect population profiles based on the dimensions' variability of burnout, as shown in previous studies (Lyndon et al., 2017). Now, considering the main findings of the reviewed studies and the scarcity of works that address the effect that compassion toward others and self‐compassion have on burnout in health‐profession students, and specifically in medical students, this research aims to meet the following research objectives.

1. Identify student profiles based on the depersonalization, emotional exhaustion, and personal accomplishment dimensions that characterize burnout syndrome.
2. Establish differences in the levels of compassion toward others and self‐compassion based on each profile.
3. Identify the differential effect that compassion toward others and self‐compassion produce in the three dimensions of burnout in each profile.

## METHOD

### Participants and procedure

The sample is made up of 235 medical students (61.7% women) from a private university, located in the Colombian Caribbean region, aged between 16 and 32 years old (*M* = 19.82; *SD* = 2.37), who were studying in the second (*n* = 181) and the fifth (*n* = 54) year out of the six years that the degree lasts in total. The students signed an informed consent form and anonymously answered a self‐administered questionnaire. Field work was carried out between February and April of 2020. The research protocol was approved by the Universidad Pontificia Bolivariana Ethics Committee and was recorded in act no. 18 with identification number 245M‐07/19‐G003.

### Instruments

To assess burnout, the version of the Maslach Burnout Inventory (Maslach et al., 1997) adapted to the Colombian population (Córdoba et al., 2012) was used. The instrument consists of 22 items evaluated on a Likert scale, with 7 response options based on the frequency with which certain sensations related to work are experienced (e.g., “*At the end of the day I feel exhausted*”). The instrument evaluates the emotional exhaustion dimension with nine items; the depersonalization dimension with five items; and the personal accomplishment dimension with eight items. This last dimension, unlike in other versions of the MBI that evaluate the lack of personal accomplishment, in the adaptation to the Colombian population measures personal accomplishment, so this dimension will tend to be inversely related to the emotional exhaustion and depersonalization factors, without altering the essence of the theoretical model. In this study, the instrument showed acceptable reliability indices according to psychometric standards both for the emotional exhaustion dimension (α = .77) and for the personal accomplishment scale (α = .72). The depersonalization dimension yielded internal consistency values slightly lower than the standard reliability values (α = .56). However, psychometric studies (Nunnally & Bernstein, 1994) consider Cronbach's alpha index values greater than .50 to be adequate.

To assess compassion toward others and self‐compassion, an instrument developed by Gilbert et al. (2017) was applied. To evaluate each type of compassion, 13 items evaluated on a Likert scale, with 11 response options based on frequency (0 = *never*; 10 = *always*) were used. The reliability of both subscales presented optimal psychometric properties (α = .77 in the compassion toward others dimension and α = .75 in the self‐compassion dimension). An example of an item from the self‐compassion scale is “*I commit to and manage my suffering when it appears*” and one from the compassion toward others scale is “*I identify and am sensitive to the suffering of others when it appears*.” To the best of our knowledge, there are no adapted and validated instruments in the Spanish language to assess self‐compassion and compassion toward others (i.e., Elices et al., 2017); however, the scale used was originally developed by Gilbert et al. (2017) and was subsequently validated in the Colombian population by Román‐Calderón et al. (2022) who identified satisfactory psychometric properties.

### Data analysis

To identify the profiles according to the burnout dimensions (emotional exhaustion, depersonalization, and personal accomplishment), a cluster analysis was implemented using the K‐means procedure (Vuik et al., 2016). Cluster analysis is recognized as an effective technique for establishing categories or profiles according to a set of variables of interest and it is widely applied in community research (Rapkin & Luke, 1993), to segment clinical populations (McLachlan, 1992), and in health (Clatworthy et al., 2005) and counseling psychology (Borgen & Barnett, 1987), among other psychological subdisciplines. Regarding the contributions of cluster analysis to burnout research, there are several studies that have been improved by this classification method. From this perspective, Bianchi et al. (2015) applied a two‐step cluster analysis for demonstrating high levels of comorbidity between burnout and depression in a longitudinal study with a sample of French schoolteachers. A similar proposal conducted by Iacovides et al. (1999) applied cluster analysis for determining the combined prevalence of burnout and depression in nursing staff. Some recent and similar antecedents of the work presented in this paper are investigations conducted by Potard and Landais (2021) and Martínez et al. (2020) in which the authors executed cluster analysis to identify the profiles of participants according to their level of burnout. Considering the empirical evidence reviewed, cluster analysis may be a pragmatic alternative for segmenting populations according to burnout syndrome.

Following the recommendations in previous studies (i.e., Fraley & Raftery, 1998), two types of cluster analysis (K‐means and two‐step cluster analysis) were executed, with similar results obtained with respect to the number of clusters identified (*n* = 3) and regarding the distribution of participants between each group. Although, as Hardy (1996) noted, the selection of the exact number of clusters is a key decision when applying cluster analysis, our results are consonant with those obtained in previous studies that applied cluster analysis to identify profiles according to burnout syndrome (Martínez et al., 2020; Potard & Landais, 2021).

Once the participants were assigned to each profile, the nonparametric Kruskal–Wallis test (Kruskal & Wallis, 1952) was carried out because the data did not present a normal distribution. Finally, a series of linear regression analyses (18 in total) was designed to determine the effect that each type of compassion independently produces on the three burnout dimensions in each profile subsamples.

## RESULTS

Table 1 presents the descriptive statistics and the bivariate correlations between the variables examined.

**TABLE 1:** Descriptive statistics and bivariate correlations.

No.	Dimension	*M*	*SD*	1	2	3	4	5
1	Emotional exhaustion	2.85	0.99	‐				
2	Depersonalization	1.30	1.09	.412**	‐			
3	Personal accomplishment	4.37	1.01	−0.126	−.038	‐		
4	Self‐compassion	6.01	1.32	0.030	−0.106	319**	‐	
5	Compassion toward others	6.56	1.35	−0.080	−.312**	.282**	.494**	‐

** *p* < .001.

The results describe a strong association between the emotional exhaustion and depersonalization dimensions, but, contrary to theoretical expectations, an inverse relationship between the two dimensions and personal accomplishment is not identified. On the other hand, the compassion toward others and self‐compassion dimensions present a strong positive correlation, with the two types of compassion showing a complex relationship with the three burnout dimensions that includes positive and negative associations.

The first objective is to detect the profiles of medical students based on the three burnout dimensions. Table 2 shows the results of the cluster analysis applying the emotional exhaustion, depersonalization, and personal accomplishment dimensions as grouping variables.

**TABLE 2:** Distribution of cases in each cluster.

Grouping variable	Cluster 1 (*n* = 51)	Cluster 2 (*n* = 104)	Cluster 3 (*n* = 80)
Depersonalization	0.87	0.58	2.53
Emotional exhaution	2.90	2.38	3.44
Personal accomplishment	2.96	4.98	4.48

*Note*: The procedure converges in nine iterations, and the minimum distance between the initial centers of the clusters is 5.25.

The first profile (*n* = 51) is characterized by low depersonalization levels and intermediate levels of emotional exhaustion and personal accomplishment. This profile will be referred to as “*low involvement*” owing to the low personal achievement shown by the participants in this group, despite their exhibiting low levels of depersonalization and emotional exhaustion.

The second profile, the most numerous (*n* = 104), is characterized by low depersonalization levels, intermediate levels of emotional exhaustion, and high levels of personal accomplishment. This profile will be referred to as “*positive adaptation*” because the students tend to exhibit an adequate adjustment before the academic demands, and at the same time this allows them to feel fulfilled with the activities they carry out.

The third profile (*n* = 80) is characterized by intermediate depersonalization levels, medium–high levels of emotional exhaustion, and high levels of personal achievement. This profile will be referred to as “*high demand*” because although personal achievement levels are high, so are those of emotional exhaustion and depersonalization. In other words, these participants assume a high psychosocial cost for the exercise of their functions and are subjected to high stress, but at the same time their academic work translates into a considerable reward based on subjective satisfaction with their performance.

The second objective aims to establish differences in the levels of compassion toward others and self‐compassion based on each profile detected through the cluster analysis. Table 3 shows the results of the non‐parametric contrasts proposed to identify differences in the level of compassion toward others and toward oneself based on the three profiles detected.

**TABLE 3:** Nonparametric contrasts between profiles using the Kruskal–Wallis test.

Contrast variable	Profile	Average range	Kruskal–Wallis H index	*p*‐value
Self‐compassion	Low involvement (*n* = 51)	92.87	14.64	.001
	Positive adaptation (*n* = 104)	135.49		
	High demand (*n* = 80)	111.28		
Compassion toward others	Low involvement (*n* = 51)	101.09	21.13	.0001
	Positive adaptation (*n* = 104)	140.86		
	High demand (*n* = 80)	99.06		

*Note*: Degrees of freedom = 2.

Table 3 results confirm that the compassion level toward others and of self‐compassion is different for each profile, with the differences being statistically significant.

**FIGURE 1:**
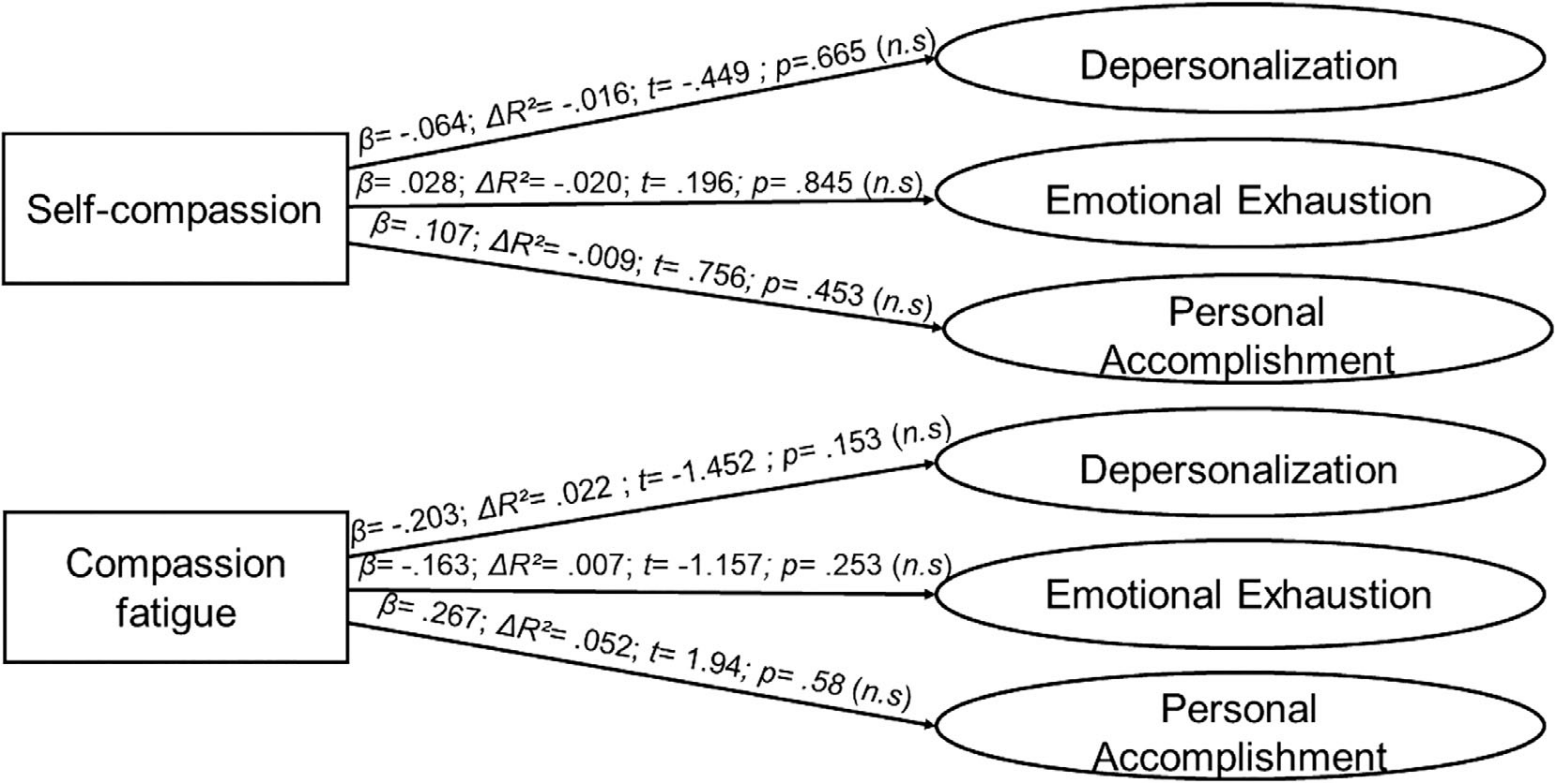
Regression analysis on the low‐involvement profile (*n* = 51). *Source*: Own elaboration.

To address the third objective, a series of linear regression analyses were carried out to discover the effect that the two compassion types have independently on each burnout dimension in the three profiles. Figure 1 illustrates the results of the linear regression analyses.

The regression analyses results reflect a differential effect of self‐compassion and compassion toward others in the three burnout dimensions, depending on each student profile. In the low‐involvement profile, no significant effects of the two types of compassion are observed in any of the burnout dimensions. These results would suggest that, for the participants in this profile, compassion is an apparently irrelevant phenomenon, with little capacity to influence the constitutive burnout dimensions.

**FIGURE 2:**
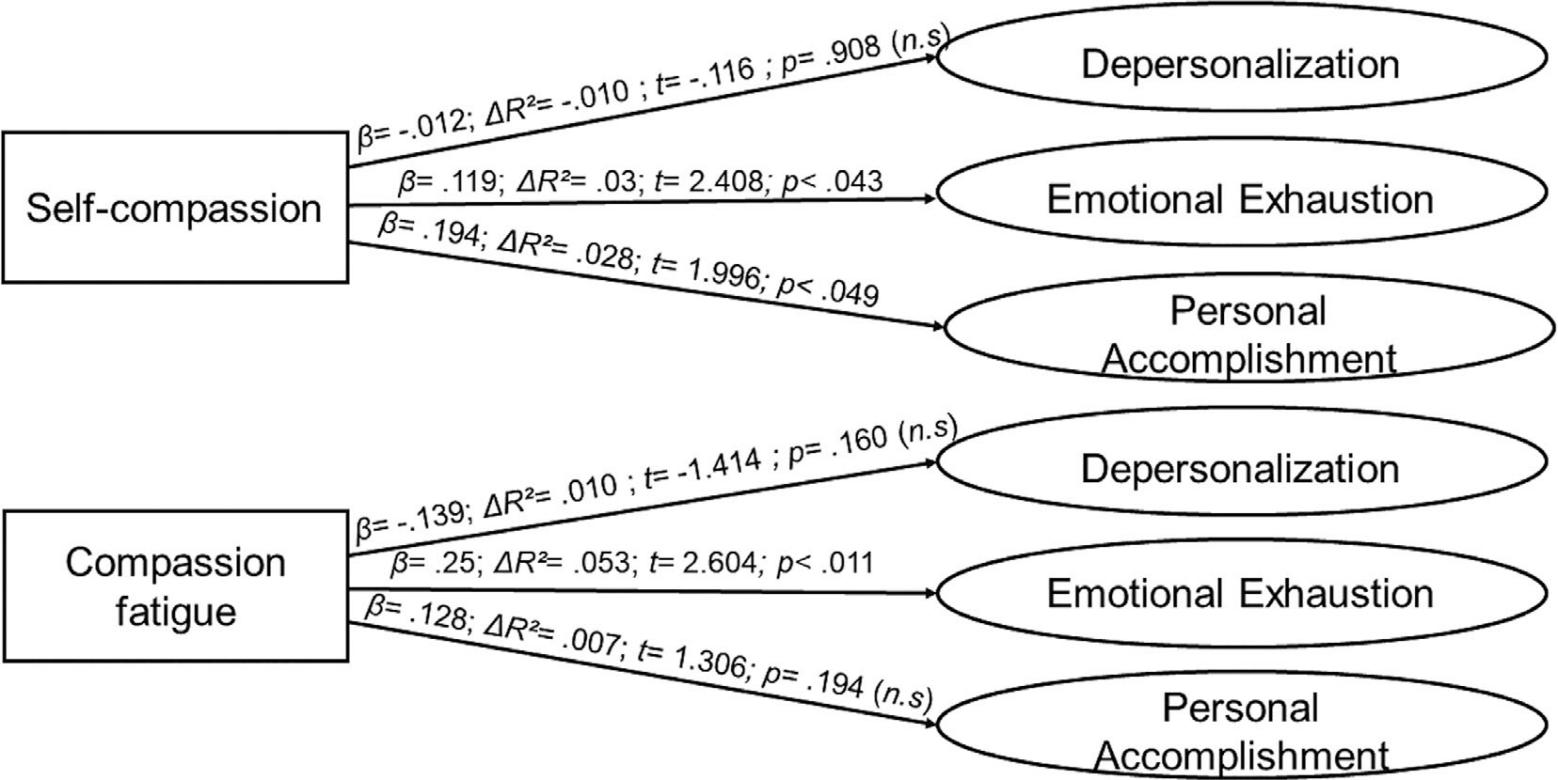
Regression analysis on the positive‐adaptation profile (*n* = 104). *Source*: Own elaboration.

**FIGURE 3:**
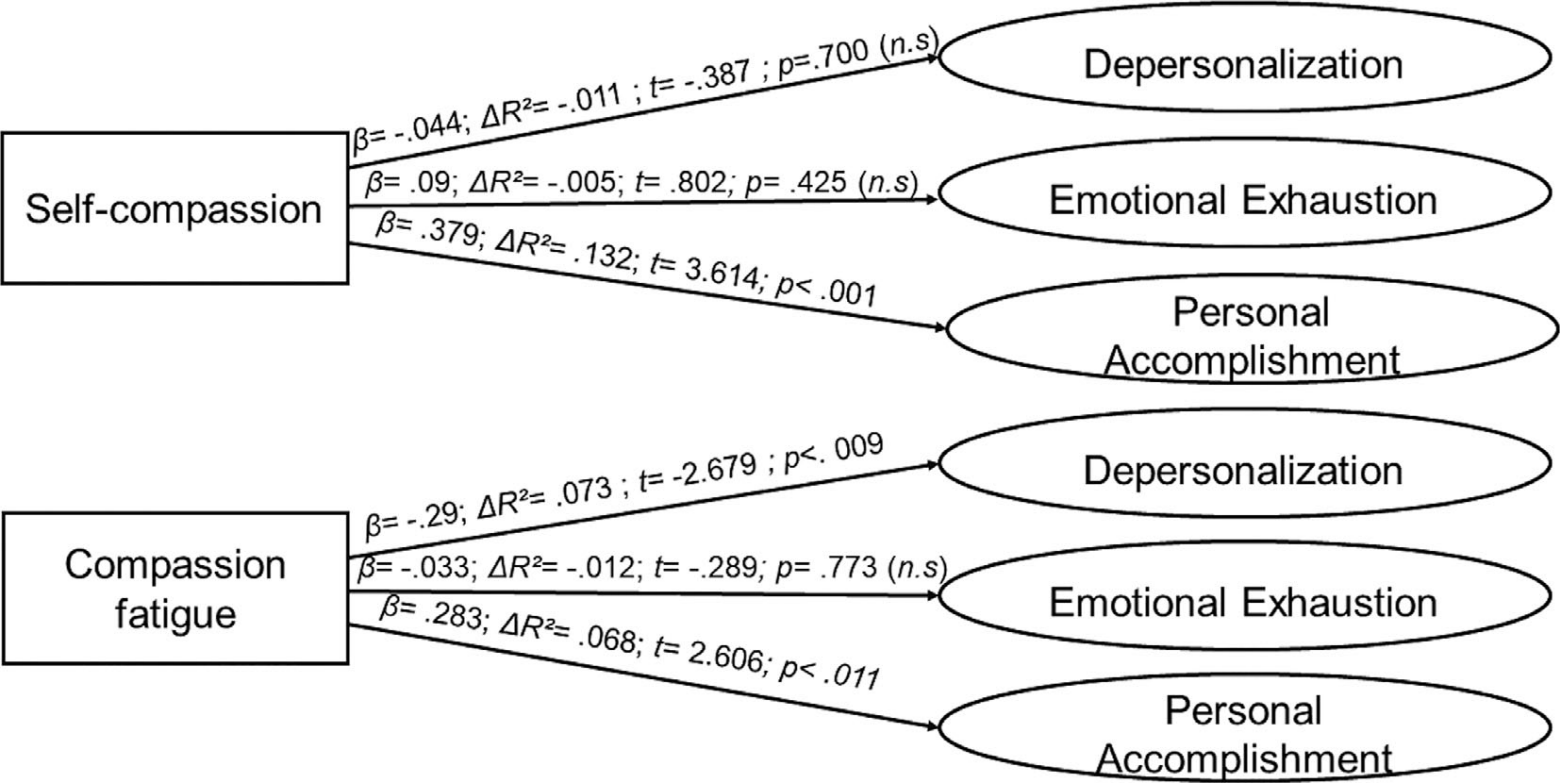
Regression analysis on the high‐demand profile (*n* = 80). *Source*: Own elaboration.

In the positive‐adaptation profile, positive and significant effects of low magnitude of self‐compassion on the emotional exhaustion and personal accomplishment dimensions are observed, but not so with respect to depersonalization. While compassion toward others is not associated with depersonalization or personal accomplishment, it has a positive and strong effect on emotional exhaustion (Figures 2 and 3).

Finally, the most disparate effects are observed in the high‐demand profile. Self‐compassion is not significatively associated with the variability of depersonalization or emotional exhaustion; however, it has a notable effect on the degree of personal accomplishment, explaining 13.2% of the variance of this dimension. For its part, compassion fatigue has negative effects of moderate intensity on depersonalization, and at the same time maintains a positive association, albeit a modest one, with personal accomplishment.

## DISCUSSION

Burnout syndrome is a public health issue that affects a large group of workers, particularly health professionals, as well as students of health specialties (Navarro‐González et al., 2015; Parola et al., 2017). The first investigations on the subject pointed out that one of the main triggers of burnout is maintaining constant contact with users (Maslach, 1978). In the case of healthcare professionals, this fact is aggravated owing to the relationship type between healthcare professionals and patients, and the wear and tear associated with the possibility of errors occurring during medical practice that can have negative consequences for the patients' health. In addition, other factors such as communication with patients and their families, transmitting unfavorable diagnoses, and the stress caused by the effort to provide a quality service are factors that put health professionals at high risk of suffering burnout throughout their career (Maslach, 1997). In the case of medical students, they share several traits with health workers in an active situation, which makes them a group that is especially susceptible to burnout (Erschens et al., 2019; Madigan & Curran, 2021).

There are some circumstances that may explain the kinds of profiles identified according to burnout dimensions. Features of the low‐involvement profile may be partially due to participants not developing an active behavior in their academic and professional activity, which translates into low levels of emotional exhaustion and depersonalization feelings, but which, at the same time, leads to a perception of low personal accomplishment. In the case of the positive‐adaptation profile, participants of this group face the stressors of their activity in such a way that they manage to respond to the demands that their activity entails, and this translates into a high level of personal accomplishment, which constitutes a positive indicator of psychosocial adaptation in the academic context. It could be said that this profile is the most desirable insofar as it implies a positive response to demands in the academic context. Finally, regarding the high‐demand profile, it is possible that the high personal accomplishment is compensated by the high levels of exhaustion and depersonalization, although it is foreseeable that in the long term it will be difficult to maintain this balance.

Active professionals and medical students are no strangers to patients' circumstances, particularly in cases where they witness their suffering as a result of disease or the treatment prescribed to alleviate it. This fact has been considered a form of vicarious trauma that causes effects similar to those caused by post‐traumatic stress disorder in mental health of the population (Figley, 1995; McCann & Pearlmann, 1990).

Medical students live with the suffering of patients, and in many cases this generates empathy as they become aware of the suffering of others, which in turn activates cognitive, emotional, and affective mechanisms that translate into feelings of compassion toward oneself and others (Figley, 1995; Neff, 2003). The few studies found that examine the effects that self‐compassion and compassion toward others have on burnout (Beaumont et al., 2016; Michalec et al., 2013) seem to indicate that self‐compassion acts as a protective factor against burnout, while compassion toward others tends to increase the syndrome.

The results of this research study show differential results based on the three profiles detected, which allowed the participants to be segmented according to burnout dimensions. These findings suggest that the two compassion types are to a certain extent inconsequential in students who present a moderate level of emotional exhaustion that it is not associated with their depersonalization level, and at the same time they feel low accomplishment. In the positive‐adaptation profile, we found a result that slightly contradicts the theoretical expectation, namely that self‐compassion, instead of reducing the negative symptoms of burnout, tends to increase the depersonalization level. This result may indicate that in students with low levels of depersonalization and emotional exhaustion, feeling compassion toward oneself when observing the suffering of others can aggravate the depersonalization level. A plausible explanation for this can be found in the fact that self‐compassion can increase burnout in cases in which professionals feel accomplished at work despite witnessing the suffering of others, which could translate into feelings of guilt or shame for exhibiting a self‐compassionate feeling while being aware that the patient is the one who truly suffers (Raab, 2014). The relationship between both compassion types and burnout dimensions in students who feel very depersonalized and exhausted because of work becomes more complex, but at the same time they manage to feel fulfilled. In the high‐demand profile, findings confirm the results reported in previous studies carried out both with health professionals and with students of health specialties (Michalec et al., 2013; Zhang et al., 2018). These studies suggest that self‐compassion produces positive effects on personal accomplishment and is also the most intense effect.

In the low‐involvement profile, the self‐compassion level is the lowest, something that may be due to the low level of involvement and personal accomplishment that academic activity seems to entail (Beaumont et al., 2016). Conversely, in the other two profiles, particularly in the positive‐adaptation profile, the self‐compassion level is the highest, which may indicate that the compassion feeling, toward both oneself and others, may be a relevant factor in the contextual, academic, and situational adaptation process (Galiana et al., 2022). In the case of the high‐demand profile, it is observed that compassion, particularly that referred to oneself, is an especially salient characteristic. These findings indicate that there are substantive variations in the degree of self‐compassion and compassion fatigue in the profiles identified according to the dimensions of burnout.

The findings of this work have several implications at both theoretical and practical levels. This study advances an understanding of how compassion toward oneself exerts differential effects on medical students’ mental health depending on the level of implication and commitment toward care activities that participants should develop during their formative stage. Self‐compassion seems to be less relevant to explaining variability in burnout dimensions when students show low involvement in providing care to patients. Conversely, when participants are highly involved/responsible or perceive an elevated level of demand, the associations of self‐compassion with burnout dimensions are increased, particularly in the personal accomplishment dimension. These results are in line with previous studies suggesting that self‐compassion could produce differential effects on mental health depending on the degree of students’ involvement in patient‐care tasks (Coaston, 2017).

With respect to compassion fatigue, our results suggest that it seems to produce no effects on burnout dimensions in medical students with low involvement levels in patient‐care activities. It is possible that this could be partially explained by the fact that these participants perceive that they are not on the frontline of patient care. In contrast, in highly involved participants, compassion fatigue produces notable effects on emotional exhaustion, while in students who perceive high demands, compassion fatigue is associated with depersonalization and personal accomplishment. This phenomenon may be explained by the level of concern experienced by medical students in the context of patient–professional interactions. Less involved participants may tend to distance themselves from patient suffering; in this case, compassion toward others will show less incidence on burnout. For participants who are strongly connected with patients during care provision, however, feelings of empathy could be exacerbated, thereby increasing psychological distress and consequently burnout symptoms, as demonstrated in previous studies conducted with nursing staff during the COVID‐19 outbreak (Ruiz‐Fernández et al., 2020).

At an application level, our findings endorse the importance of segmenting populations when designing and implementing interventions to reduce psychosocial work‐related risk factors and to promote effective coping styles to address the effects of compassion fatigue. Designing intervention strategies according to the level of burnout of medical students could have various positive impacts. First, the cost of interventions can be reduced through the segmentation process by allowing the detection of subgroups that can really benefit from a program, for example from mindfulness interventions to reduce compassion fatigue (Clarkson et al., 2019). Second, identifying those medical students who are at risk of suffering severe mental health problems because of their burnout levels due to compassion fatigue means that these students can be prioritized in the intervention process. Finally, knowledge of the exact characteristics of participants according to their burnout level means that it is possible to adapt intervention content, intensity, and dosage according to the real needs of each subpopulation. In line with this, some studies suggest that segmenting populations is an effective strategy for increasing access to interventions and for reaching populations with specific needs (e.g., Evans, 2016).

The stage of the training cycle at which interventions aimed at reducing the harmful effects of burnout should be implemented is a key decision. In this regard, a proposal developed by Tucker et al. (2017) suggested that the third year of training is the time to apply interventions aimed at managing feelings of compassion and at reducing the damage caused by burnout syndrome. The authors suggested that the third year of training is the moment at which a transition from theory to practice occurs, which is accompanied by an increase in contact with patients, which can be considered a psychosocial stressor for health professionals (p. 110). At the same time, the same study highlighted the need to be attentive to subtle signs that may denote problems managing feelings related to compassion and the characteristic symptoms of burnout. Previous studies (e.g., Duarte & Pinto‐Gouveia, 2017; Figley, 2002) suggest that personal distress, psychological inflexibility, decreased levels of satisfaction, excessive feelings of guilt, or abrupt changes in behavior with colleagues and users are signs that anticipate the appearance of the harmful effects of both compassion fatigue and burnout.

In essence, the results of this research show that self‐compassion and compassion toward others, feelings commonly experienced by those who work in the medical professions and who study to become health professionals, have differential associations with burnout. In this sense, compassion fatigue tends to aggravate burnout, so it is recommended that programs should be implemented so that students learn to positively identify, manage, and regulate feelings of compassion toward themselves and others from the first years of their health careers, but particularly in the transition from theory to practice, which tends to occur in the third year (Tucker et al., 2017). Finally, normalizing the experience of compassion fatigue and burnout syndrome in medical students could have several positive consequences, including the early detection of symptoms and increased self‐care practices that have been shown to be effective in reducing harm and increasing satisfaction and psychosocial well‐being (Harr, 2013).

There are some limitations that must be explained. The design of this research is cross‐sectional, so although correlations can be established, it is not possible to establish causal relationships between the compassion types and burnout dimensions. Second, the analyses include students from the first and last years of their career studies, and there is also an evident imbalance when representing the two subpopulations; thus, it is possible that the differential experience based on the years they have been studying may be conditioning the results. Third, the students belong to a single educational institution, so the institutional policies and cultural context could determine the way in which the residencies are developed and the subsequent contact with the patients, factors that in turn could affect the relationship between the examined variables. Finally, we must note that the depersonalization dimension of MBI exhibits poor psychometric properties (α = .56) compared with the other scales applied in this study, which present Cronbachs' alpha values above .70, which is considered an acceptable threshold in psychometric research (Oviedo & Campo‐Arias, 2005). Some authors suggest that the reliability and internal consistency of instruments is, at least partially, related to the number of items and the item response format (Cortina, 1993, pp. 100–101). In the case of depersonalization, this dimension includes fewer items (five) than the nine that evaluate emotional exhaustion and the eight that evaluate personal accomplishment. According to Cortina (1993), this may be a plausible explanation for the low internal consistency detected in this subscale.

## FUNDING INFORMATION

Research project: “Relación entre empatía y burnout en estudiantes de medicina y profesionales médicos de dos ciudades de Colombia” (245M‐07/19‐G003). This initiative is supported by the Universidad Pontificia Bolivariana.

## CONFLICT OF INTEREST STATEMENT

The authors declare there are no conflicts of interest.

## ETHICS STATEMENT

The research protocol was approved by the Universidad Pontificia Bolivariana ethics committee, and recorded in act no. 18 with identification number 245M‐07/19‐G003.

## Data Availability

The data analyzed in this research are available upon request to the authors.

## References

[pchj692-bib-0001] Beaumont, E. , Durkin, M. , Hollins, C. J. , & Carson, J. (2016). Measuring relationships between self‐compassion, compassion fatigue, burnout and well‐being in student counsellors and student cognitive behavioral psychotherapists: A quantitative survey. Counselling and Psychotherapy Research, 16(1), 15–23. 10.1002/capr.12054

[pchj692-bib-0003] Bianchi, R. , Schonfeld, I. S. , & Laurent, E. (2015). Is burnout separable from depression in cluster analysis? A longitudinal study. Social Psychiatry and Psychiatric Epidemiology, 50, 1005–1011. 10.1007/s00127-014-0996-8 25527209

[pchj692-bib-0004] Boni, R. A. D. S. , Paiva, C. E. , De Oliveira, M. A. , Lucchetti, G. , Fregnani, J. H. T. G. , & Paiva, B. S. R. (2018). Burnout among medical students during the first years of undergraduate school: Prevalence and associated factors. PLoS One, 13(3), e0191746. 10.1371/journal.pone.0191746 29513668 PMC5841647

[pchj692-bib-0005] Borgen, F. H. , & Barnett, D. C. (1987). Applying cluster analysis in counseling psychology research. Journal of Counseling Psychology, 34(4), 456–468. 10.1037/0022-0167.34.4.456

[pchj692-bib-0006] Caballero, C. , Hederich, C. , & Palacio, J. (2010). El burnout académico: delimitación del síndrome y factores asociados a su aparición. Revista Latinoamericana Psicología, 42(1), 131–146.

[pchj692-bib-0007] Clarkson, M. , Heads, G. , Hodgson, D. , & Probst, H. (2019). Does the intervention of mindfulness reduce levels of burnout and compassion fatigue and increase resilience in pre‐registration students? A pilot study. Radiography, 25(1), 4–9. 10.1016/j.radi.2018.08.003 30599829

[pchj692-bib-0008] Clatworthy, J. , Buick, D. , Hankins, M. , Weinman, J. , & Horne, R. (2005). The use and reporting of cluster analysis in health psychology: A review. British Journal of Health Psychology, 10(3), 329–358. 10.1348/135910705X25697 16238852

[pchj692-bib-0009] Coaston, S. C. (2017). Self‐care through self‐compassion: A balm for burnout. Professional Counselor, 7(3), 285–297. 10.15241/SCC.7.3.285

[pchj692-bib-0010] Córdoba, L. , Tamayo, J. A. , González, M. A. , Martínez, M. I. , Rosales, A. , & Barbato, S. H. (2012). Adaptation and validation of the Maslach burnout inventory‐human services survey in Cali, Colombia. Colombia Médica, 42(3), 286–293.

[pchj692-bib-0076] Cortina, J. M . (1993). What is coefficient alpha? An examination of theory and applications. Journal of applied psychology, 78(1), 98–104.

[pchj692-bib-0011] Dahlin, M. E. , & Runeson, B. (2007). Burnout and psychiatric morbidity among medical students entering clinical training: A three year prospective questionnaire and interview‐based study. BMC Medical Education, 7, 1–8. 10.1186/1472-6920-7-6 17430583 PMC1857695

[pchj692-bib-0013] Duarte, J. , & Pinto‐Gouveia, J. (2017). The role of psychological factors in oncology nurses' burnout and compassion fatigue symptoms. European Journal of Oncology Nursing, 28, 114–121. 10.1016/j.ejon.2017.04.002 28478848

[pchj692-bib-0014] Elices, M. , Carmona, C. , Pascual, J. C. , Feliu‐Soler, A. , Martin‐Blanco, A. , & Soler, J. (2017). Compassion and self‐compassion: Construct and measurement. Mindfulness & Compassion, 2(1), 34–40. 10.1016/j.mincom.2016.11.003

[pchj692-bib-0015] Erschens, R. , Keifenheim, K. E. , Herrmann‐Werner, A. , Loda, T. , Schwille‐Kiuntke, J. , Bugaj, T. J. , Nikendei, C. , Huhn, D. , Zipfel, S. , & Junne, F. (2019). Professional burnout among medical students: Systematic literature review and meta‐analysis. Medical Teacher, 41(2), 172–183. 10.1080/0142159X.2018.1457213 29656675

[pchj692-bib-0016] Evans, W. D. (2016). Social marketing research for global public health: Methods and technologies. Oxford University Press.

[pchj692-bib-0017] Faye‐Dumanget, C. , Carré, J. , Le Borgne, M. , & Boudo Ukha, P. A. H. (2017). French validation of the Maslach burnout inventory‐student survey (MBI‐SS). Journal of Evaluation in Clinical Practice, 23(6), 1247–1251. 10.1111/jep.12771 28653800

[pchj692-bib-0018] Fernández‐Suárez, I. , García‐González, M. A. , Torrano, F. , & García‐González, G. (2021). Study of the prevalence of burnout in university professors in the period 2005–2020. Education Research International, 7810659, 1–10. 10.1155/2021/7810659

[pchj692-bib-0019] Figley, C. R. (Ed.). (1995). Compassion fatigue: Coping with secondary traumatic stress disorder. Brunner/Mazel.

[pchj692-bib-0020] Figley, C. R. (Ed.). (2002). Treating compassion fatigue. Brunner/Routledge.

[pchj692-bib-0021] Frajerman, A. , Morvan, Y. , Krebs, M. O. , Gorwood, P. , & Chaumette, B. (2019). Burnout in medical students before residency: A systematic review and meta‐analysis. European Psychiatry, 55, 36–42. 10.1016/j.eurpsy.2018.08.006 30384110

[pchj692-bib-0022] Fraley, C. , & Raftery, A. E. (1998). How many clusters? Which clustering method? Answers via model‐based cluster analysis. The Computer Journal, 41(8), 578–588. 10.1093/comjnl/41.8.578

[pchj692-bib-0023] Freudenberger, H. J. (1974). Staff burn‐out. Journal of Social Issues, 30(1), 159–165. 10.1111/j.1540-4560.1974.tb00706.x

[pchj692-bib-0024] Galiana, L. , Sansó, N. , Muñoz‐Martínez, I. , Vidal‐Blanco, G. , Oliver, A. , & Larkin, P. J. (2022). Palliative care professionals' inner life: Exploring the mediating role of self‐compassion in the prediction of compassion satisfaction, compassion f atigue, burnout and wellbeing. Journal of Pain and Symptom Management, 63(1), 112–123. 10.1016/j.jpainsymman.2021.07.004 34271144

[pchj692-bib-0025] Gerber, Z. , & Anaki, D. (2021). The role of self‐compassion, concern for others, and basic psychological needs in the reduction of caregiving burnout. Mindfulness, 12, 741–750. 10.1007/s12671-020-01540-1 33224308 PMC7667216

[pchj692-bib-0026] Gilbert, P. (2009). The compassionate mind. Constable.

[pchj692-bib-0027] Gilbert, P. , Catarino, F. , Duarte, C. , Matos, M. , Kolts, R. , Stubbs, J. , Ceresatto, L. , Duarte, J. , Pinto‐Gouveia, J. , & Basran, J. (2017). The development of compassionate engagement and action scales for self and others. Journal of Compassionate Health Care, 4(1), 1–24. 10.1186/s40639-017-0033-3

[pchj692-bib-0029] Hardy, A. (1996). On the number of clusters. Computational Statistics & Data Analysis, 23(1), 83–96. 10.1016/S0167-9473(96)00022-9

[pchj692-bib-0030] Harr, C. (2013). Promoting workplace health by diminishing the negative impact of compassion fatigue and increasing compassion satisfaction. Social Work and Christianity, 40(1), 71–88.

[pchj692-bib-0031] Hashem, Z. , & Zeinoun, P. (2020). Self‐compassion explains less burnout among healthcare professionals. Mindfulness, 11, 2542–2551. 10.1007/s12671-020-01469-5 32929384 PMC7481342

[pchj692-bib-0032] Iacovides, A. , Fountoulakis, K. N. , Moysidou, C. , & Ierodiakonou, C. (1999). Burnout in nursing staff: Is there a relationship between depression and burnout? The International Journal of Psychiatry in Medicine, 29(4), 421–433. 10.2190/5YHH-4CVF-99M4-MJ28 10782425

[pchj692-bib-0033] Jiménez‐Gómez, L. , Yela, J. R. , Crego, A. , Melero‐Ventola, A. R. , & Gómez‐Martínez, M. Á. (2022). Effectiveness of the mindfulness‐based stress reduction (MBSR) vs. the mindful self‐compassion (MSC) programs in clinical and health psychologist trainees. Mindfulness, 13(3), 584–599. 10.1007/s12671-021-01814-2

[pchj692-bib-0034] Kemper, K. J. , McClafferty, H. , Wilson, P. M. , Serwint, J. R. , Batra, M. , Mahan, J. D. , & Pediatric Resident Burnout‐Resilience Study Consortium . (2019). Do mindfulness and self‐compassion predict burnout in pediatric residents? Academic Medicine, 94(6), 876–884. 10.1097/ACM.0000000000002546 30520809

[pchj692-bib-0035] Klein, C. J. , Riggenbach‐Hays, J. J. , Sollenberger, L. M. , Harney, D. M. , & McGarvey, J. S. (2018). Quality of life and compassion satisfaction in clinicians: A pilot intervention study for reducing compassion fatigue. American Journal of Hospice and Palliative Medicine®, 35(6), 882–888. 10.1177/1049909117740848 29169248

[pchj692-bib-0036] Kocalevent, R. , Pinnschmidt, H. , Selch, S. , Nehls, S. , Meyer, J. , Boczor, S. , Scherer, M. , & van den Bussche, H. (2020). Burnout is associated with work‐family conflict and gratification crisis among German resident physicians. BMC Medical Education, 20(1), 1–8. 10.1186/s12909-020-02061-0 PMC7206716 32384889

[pchj692-bib-0037] Kruskal, W. H. , & Wallis, W. A. (1952). Use of ranks in one‐criterion variance analysis. Journal of the American Statistical Association, 47(260), 583–621. 10.1080/01621459.1952.10483441

[pchj692-bib-0038] Kusurkar, R. A. , van der Burgt, S. M. , Isik, U. , Mak‐van der Vossen, M. , Wilschut, J. , Wouters, A. , & Koster, A. S. (2021). Burnout and engagement among PhD students in medicine: The BEeP study. Perspectives on Medical Education, 10, 110–117. 10.1007/s40037-020-00637-6 33284408 PMC7952475

[pchj692-bib-0039] Lyndon, M. P. , Henning, M. A. , Alyami, H. , Krishna, S. , Zeng, I. , Yu, T. C. , & Hill, A. G. (2017). Burnout, quality of life, motivation, and academic achievement among medical students: A person‐oriented approach. Perspectives on Medical Education, 6(2), 108–114. 10.1007/s40037-017-0340-6 28247209 PMC5383573

[pchj692-bib-0040] Madigan, D. J. , & Curran, T. (2021). Does burnout affect academic achievement? A meta‐analysis of over 100,000 students. Educational Psychology Review, 33(2), 387–405. 10.1007/s10648-020-09533-1

[pchj692-bib-0042] Martínez, J. P. , Méndez, I. , Ruiz‐Esteban, C. , Fernández‐Sogorb, A. , & García‐Fernández, J. M. (2020). Profiles of burnout, coping strategies and depressive symptomatology. Frontiers in Psychology, 11, 591. 10.3389/fpsyg.2020.00591 32300323 PMC7142211

[pchj692-bib-0043] Martínez‐Rubio, D. , Martínez‐Brotons, C. , Monreal‐Bartolomé, A. , Barceló‐Soler, A. , Campos, D. , Pérez‐Aranda, A. , Colomer‐Carbonell, A. , Cervera‐Torres, S. , Solé, S. , Moreno, Y. , & Montero‐Marín, J. (2021). Protective role of mindfulness, self‐compassion and psychological flexibility on the burnout subtypes among psychology and nursing undergraduate students. Journal of Advanced Nursing, 77(8), 3398–3411. 10.1111/jan.14870 33905551

[pchj692-bib-0044] Maslach, C. (1978). The client role in staff burn‐out. Journal of Social Issues, 34(4), 111–124. 10.1111/j.1540-4560.1978.tb00778.x

[pchj692-bib-0045] Maslach, C. (1997). Burnout in health professionals. In Cambridge handbook of psychology, health and medicine (pp. 427–430). Cambridge University Press.

[pchj692-bib-0046] Maslach, C. , & Jackson, S. E. (1981). The Maslach burnout inventory. Consulting Psychologists Press.

[pchj692-bib-0047] Maslach, C. , Jackson, S. E. , & Leiter, M. P. (1997). Maslach burnout inventory. Scarecrow Education.

[pchj692-bib-0048] Maslach, C. , & Leiter, M. P. (2016). Burnout. In Stress: Concepts, cognition, emotion, and behavior (pp. 351–357). Academic Press.

[pchj692-bib-0049] Maslach, C. , & Schaufeli, W. B. (2018). Historical and conceptual development of burnout. In Professional burnout: Recent developments in theory and research (pp. 1–16). CRC Press.

[pchj692-bib-0050] McCann, I. L. , & Pearlmann, L. A. (1990). Vicarious traumatization: A framework for understanding the psychological effects of working with victims. Journal of Traumatic Stress, 3, 131–149. 10.1007/BF00975140

[pchj692-bib-0051] McLachlan, G. J. (1992). Cluster analysis and related techniques in medical research. Statistical Methods in Medical Research, 1(1), 27–48. 10.1177/096228029200100103 1341650

[pchj692-bib-0052] Michalec, B. , Diefenbeck, C. , & Mahoney, M. (2013). The calm before the storm? Burnout and compassion fatigue among undergraduate nursing students. Nurse Education Today, 33(4), 314–320. 10.1016/j.nedt.2013.01.026 23434192

[pchj692-bib-0053] Montero‐Marin, J. , Zubiaga, F. , & Cereceda, M. (2020). Correction: Burnout subtypes and absence of self‐compassion in primary healthcare professionals: A cross‐sectional study. PLoS One, 15(4), e0231370. 10.1371/journal.pone.0157499 32236141 PMC7112209

[pchj692-bib-0054] Navarro‐González, D. , Ayechu‐Díaz, A. , & Huarte‐Labiano, I. (2015). Prevalencia del síndrome del burnout y factores asociados a dicho síndrome en los profesionales sanitarios de Atención Primaria. SEMERGEN‐Medicina de Familia, 41(4), 191–198. 10.1016/j.semerg.2014.03.008 24857630

[pchj692-bib-0055] Neff, K. (2003). Self‐compassion: An alternative conceptualization of a healthy attitude toward oneself. Self and Identity, 2(2), 85–101. 10.1080/15298860309032

[pchj692-bib-0056] Nunnally, B. , & Bernstein, I. R. (1994). Psychometric theory. Oxford University.

[pchj692-bib-0057] Ondrejková, N. , & Halamová, J. (2022). Prevalence of compassion fatigue among helping professions and relationship to compassion for others, self‐compassion and self‐criticism. Health & Social Care in the Community, 30(5), 1680–1694. 10.1111/hsc.13741 35133041

[pchj692-bib-0058] Ortega‐Campos, E. , Vargas‐Román, K. , Velando‐Soriano, A. , Suleiman‐Martos, N. , Cañadas‐de la Fuente, G. A. , Albendín‐García, L. , & Gómez‐Urquiza, J. L. (2019). Compassion fatigue, compassion satisfaction, and burnout in oncology nurses: A systematic review and meta‐analysis. Sustainability, 12(1), 72. 10.3390/su12010072

[pchj692-bib-0059] Oviedo, H. C. , & Campo‐Arias, A. (2005). Aproximación al uso del coeficiente alfa de Cronbach. Revista Colombiana de Psiquiatría, 34(4), 572–580.

[pchj692-bib-0060] Parola, V. , Coelho, A. , Cardoso, D. , Sandgren, A. , & Apóstolo, J. (2017). Prevalence of burnout in health professionals working in palliative care: A systematic review. JBI Evidence Synthesis, 15(7), 1905–1933. 10.11124/JBISRIR-2016-00330 28708752

[pchj692-bib-0061] Pereira, A. T. , Brito, M. J. , Cabaços, C. , Carneiro, M. , Carvalho, F. , Manão, A. , Araújo, A. , Pereira, D. , & Macedo, A. (2022). The protective role of self‐compassion in the relationship between perfectionism and burnout in Portuguese medicine and dentistry students. International Journal of Environmental Research and Public Health, 19(5), 2740. 10.3390/ijerph19052740 35270432 PMC8910448

[pchj692-bib-0062] Potard, C. , & Landais, C. (2021). The use of cluster analysis to identify different burnout profiles among nurses and care assistants for older adults. Geriatric Nursing, 42(5), 1135–1142. 10.1016/j.gerinurse.2021.07.005 34352680

[pchj692-bib-0063] Raab, K. (2014). Mindfulness, self‐compassion, and empathy among health care professionals: A review of the literature. Journal of Health Care Chaplaincy, 20(3), 95–108. 10.1080/08854726.2014.913876 24926896

[pchj692-bib-0064] Rapkin, B. D. , & Luke, D. A. (1993). Cluster analysis in community research: Epistemology and practice. American Journal of Community Psychology, 21(2), 247–277. 10.1007/BF00941623

[pchj692-bib-0065] Richardson, M. , Abraham, C. , & Bond, R. (2012). Psychological correlates of university students' academic performance: A systematic review and meta‐analysis. Psychological Bulletin, 138(2), 353–387. 10.1037/a0026838 22352812

[pchj692-bib-0066] Román‐Calderón, J. P. , Krikorian, A. , Ruiz, E. , Romero, A. M. , & Lemos, M. (2022). Compassion and self‐compassion: Counterfactors of burnout in medical students and physicians. Psychological Reports. 10.1177/00332941221132995 36219581

[pchj692-bib-0067] Ruiz‐Fernández, M. D. , Ramos‐Pichardo, J. D. , Ibáñez‐Masero, O. , Cabrera‐Troya, J. , Carmona‐Rega, M. I. , & Ortega‐Galán, Á. M. (2020). Compassion fatigue, burnout, compassion satisfaction and perceived stress in healthcare professionals during the COVID‐19 health crisis in Spain. Journal of Clinical Nursing, 29(21–22), 4321–4330. 10.1111/jocn.15469 32860287

[pchj692-bib-0068] Spickard, A., Jr. , Gabbe, S. G. , & Christensen, J. F. (2002). Mid‐career burnout in generalist and specialist physicians. Jama, 288(12), 1447–1450. 12243624 10.1001/jama.288.12.1447

[pchj692-bib-0069] Tucker, T. , Bouvette, M. , Daly, S. , & Grassau, P. (2017). Finding the sweet spot: Developing, implementing and evaluating a burn out and compassion fatigue intervention for third year medical trainees. Evaluation and Program Planning, 65, 106–112. 10.1016/j.evalprogplan.2017.07.006 28763733

[pchj692-bib-0071] Vuik, S. I. , Mayer, E. , & Darzi, A. (2016). A quantitative evidence base for population health: Applying utilization‐based cluster analysis to segment a patient population. Population Health Metrics, 14(1), 1–9. 10.1186/s12963-016-0115-z 27906004 PMC5124281

[pchj692-bib-0072] Wang, M. , Guan, H. , Li, Y. , Xing, C. , & Rui, B. (2019). Academic burnout and professional self‐concept of nursing students: A cross‐sectional study. Nurse Education Today, 77, 27–31. 10.1016/j.nedt.2019.03.004 30939399

[pchj692-bib-0074] Zhang, Y. Y. , Han, W. L. , Qin, W. , Yin, H. X. , Zhang, C. F. , Kong, C. , & Wang, Y. L. (2018). Extent of compassion satisfaction, compassion fatigue and burnout in nursing: A meta‐analysis. Journal of Nursing Management, 26(7), 810–819. 10.1111/jonm.12589 30129106

